# An insight into the estimation of frost thermal conductivity on parallel surface channels using kernel based GPR strategy

**DOI:** 10.1038/s41598-021-86607-2

**Published:** 2021-03-30

**Authors:** Xuejun Zhou, Fangyuan Zhou, Maryam Naseri

**Affiliations:** 1 College of Physics and Electronic Information, Yan’an University, Yan’an, 716000 Shaanxi China; 2 Department of Chemical Engineering, Faculty of Engineering, Golestan University, Aliabad Katoul, Iran

**Keywords:** Chemistry, Energy science and technology, Engineering, Mathematics and computing, Nanoscience and technology

## Abstract

In heat exchange applications, frost formation on the cold surface causes a decrease in the rate of heat transfer and growth in the pressure drop. Thus, the study on the frost thermal conductivity has a significant and vital place for the engineers and researchers dealing with the heat exchangers. In the literature, there is a lack of accurate and applicable methods for determination of frost thermal conductivity. Additionally, the high cost and difficulties of experimental works clarify the importance of computational and mathematical methods. The errors in the determination of frost thermal conductivity on parallel surface channels can cause inaccuracy in estimations of frost density and thickness. The main aim of present work is suggesting Gaussian Process Regression (GPR) models based on four different kernel functions for the estimation of frost thermal conductivity in terms of time, air velocity, relative humidity, air temperature, wall temperature, and frost porosity. To achieve this purpose, a total number of 57 frost thermal conductivity values has been collected. Comparing the suggested GPR models and other available computational methods express the quality of the developed models. The best predictive tool has been selected as a GPR model, including Matern kernel function with R^2^ values of 0.997 and 0.994 in training and testing phases, respectively. In addition, the effectiveness of discussing variables on frost thermal conductivity has been investigated by sensitivity analysis and showed that air temperature is the most effective parameter. The present work gives engineers an insight into frost thermal conductivity and the effective parameters in its determination.The significant advantage of present work is the accurate prediction of thermal conductivity by a brief knownledge in artificial intelligence.

## Introduction

As the surface temperature is lower than air dew point temperature and the water freezing temperature, moist air creates frost on a cold surface. Frost creation rate grows by increasing the air stream humidity and the temperature difference between air and the cold surface^1^. The frost deposition can be extensively happened on the surfaces of the air-source heat pump and heat exchangers in refrigeration systems, which have consequences such as increasing the pressure drop and decreasing in the heat transfer rate. Therefore, the formation of frost increase consumption of power in the refrigeration system and hear exchanger^2^.

The calculation of frost thermal conductivity, frost density, and frost thickness can be known as a challenging issue because this layer is porous media with intensive variation in porosity^3^. Although some advancements in experimental assessment of the frost layer characteristics are achieved, the laboratory investigations are time-consuming, costly, and also need more advanced equipment. Moreover, the development of a computational method can complement the measurement data for a better understanding of the impact of various variables on frost growth and thermal conductivity^4–7^.

In many heat exchangers, utilization of low-temperature parallel plates is common. Therefore, the investigation of frost on parallel plates is necessary. Additionally, it is worthy of mentioning that the frosting process on a float plate and on a parallel plate heat exchangers are different from each one. The more explanations for these differences can be found in^8^.

One of the important parameters in the formation rate of frost is the thermal conductivity of frost. Investigations in the available literature show that thermal conductivity of frost is a function of time duration, air relative humidity, wall surface temperature, air temperature, air velocity, and frost porosity. Despite difficulties, different empirical, semi-empirical, and theoretical approaches have been proposed to construct a suitable estimative tool for frost thermal conductivity determination. Generally, there are different hardships in experimental investigations for obtaining properties in different sciences^9–12^. Therefore, mathematical and computational methods have become highlighted. Yonko proposed a model for the prediction of conductivity in terms of frost density^13^. Brian et al. also suggested another model for conductivity as a function of wall temperature and frost density. Additionally, they proposed an empirical model to determine conductivity for frost densities less than 250 kg/m^3^^14^. Sanders expressed that thermal conductivity is highly a function of frost density and developed a relationship for determination of thermal conductivity with frost density less than 500 kg/m^3^^15^. Ostin et al. upgraded the Yonko model to determine conductivity for the frost density ranges of 50–680 kg/m^3^^16^. Lee et al. developed a model for the thermal conductivity by considering frost density as the most effective parameter^17^. Sturn et al. used two relationships which have limitations in some ranges of operating conditions for the prediction of frost thermal conductivity^18^. Kim proposed a relationship to determine frost thermal conductivity in terms of airflow velocity, air relative humidity, time, and frost density^19^. Negrelli took into the frost structure on the determination of the thermal conductivity over flat surfaces. Then, in the other study, wall temperature and frost porosity were considered in the calculation of the thermal conductivity^20,21^.

Among the different methods exist in the literature. Negrelli et al.^20,21^, Kim et al.^19^, Sturm et al.^18^, Lee et al.^17^, Ostin and Andersson^16^, Sanders^15^, Brian et al.^14^, and Yonko et al.^13^ models are the most popular ones in the calculation of frost thermal conductivity. Most of these investigations have been carried on frost on the flat plates in a limited range of temperature, and there is a limited number of discussing studies focuses on parallel surface channels.

As mentioned, the growth of frost on the surfaces in the heat exchanger induces a reduction in the thermal conductivity and an increase in the pressure drop. Therefore, the prediction of frost thermal conductivity has a vital importance among engineers dealing with heat exchangers. In the current study, the frost thermal conductivity on parallel surface channels has been determined by Gaussian Process Regression (GPR) methods containing four different forms of kernel functions in terms of time, air velocity, relative humidity, air temperature, wall temperature, and frost porosity. Furthermore, the comparison with real thermal conductivity for different available approaches in literature has been made to evaluate the accuracy of proposed GPR models. In addition, the sensitivity analysis for impacts of the influential variables on frost thermal conductivity has been done.

## Previous models

### Y–S model

Yonko and Sepsy proposed a model which was upgraded by other researchers^13^. This method was constructed by utilization of primary experimental data. In the aforementioned method, the thermal conductivity is obtained in terms of frost density for density ${(\rho }_{f})$ lower than 575 kg/m^3^. The Y–S model is formulated as follows:

1 $${k}_{f}=2.422\times {10}^{-2}+7.214\times {10}^{-4}{\rho }_{f}+1.1797\times {10}^{-6}{\rho }_{f}^{2}$$

### B–R–S model

Brian proposed a correlation for thermal conductivity in terms of the wall temperature and frost density^14^. The proposed model is applicable for density lower than 250 kg/m^3^. This model is described as follows:

2 $${k}_{f}=2.401\times {10}^{-5}{T}_{w}^{1.272}+3.921\times {10}^{-8}{\rho }_{f}{T}_{w}^{1.74}$$

### S model

Sanders expressed that the most important parameters for determination of the frost thermal conductivity is the frost density and suggested the below correlation^15^:

3 $${k}_{f}=1.202 \times {10}^{-3}{\rho }_{f}^{0.963}$$

### O–A model

A model was proposed by Ostin and Andersson for a density range of 50–680 kg/m^3^ as following^16^:

4 $${k}_{f}=-8.71\times {10}^{-3}+4.39\times {10}^{-4}{\rho }_{f}+1.05\times {10}^{-6}{\rho }_{f}^{2}$$

### L–L–K model

Lee et al. developed a correlation which is a function of density^17^:

5 $${k}_{f}=0.132+3.13\times {10}^{-4}{\rho }_{f}+1.6{10}^{-7}{\rho }_{f}^{2}$$

### S–H–K–M model

Sturm et al. developed below correlations for determination of the frost thermal conductivity^18^:

6 $$ \left\{ {\begin{array}{*{20}l} {k_{f} = 0.138 - 1.01 \times 10^{ - 3} \rho_{f} + 3.233 \times 10^{ - 6} \rho_{f}^{2} } \hfill & {for\;156 < \rho_{f} < 600\;{\text{kg/m}}^{3} } \hfill \\ {k_{f} = 2.3 \times 10^{ - 2} + 2.34 \times 10^{ - 4} \rho_{f} } \hfill & {for\;\rho_{f} < 156\;{\text{kg/m}}^{3} } \hfill \\ \end{array} } \right. $$

### K–J–L model

Kim et al. suggested a relationship for determination of frost thermal conductivity in terms of time, airflow velocity, air relative humidity, and frost density as following^19^:

7 $$ k_{f}  = {\frac{{8.5 \times 10^{{ - 3}} \rho _{f}^{{0.7}} t^{{0.01}} (1000\emptyset _{a} )^{{0.3}} }}{{1.66 - 0.205u_{a}  + 4.5 \times 10^{{ - 2}} u_{a}^{2} }}} $$

### N–H model

A new model was developed by Negrelli and Hermes for determination of the frost thermal conductivity, which covers three different ranges of temperature^20^. This relationship is formulated as follows:

8 $$ k_{f} = a_{1} k_{1} \left( {\frac{{k_{a} }}{{k_{i} }}} \right)^{{a_{2} \varepsilon }} $$

In which ${a}_{1}$ and ${a}_{2}$ stand for a coefficient which are determined by fitting on data.

### N–J–H model

Negrelli et al. proposed a formulation for the thermal conductivity of frost on parallel channels by assuming the impacts of frost porosity and wall temperature^21^. The thermal conductivity of the serial and parallel array are determined as below:

9 $$ {\frac{1}{{k_{s}}}} = {\frac{\varepsilon }{{k_{a} }}} + {\frac{{1 - \varepsilon }}{{k_{i} }}} $$

10 $${k}_{p}={k}_{a}\varepsilon +{k}_{i}(1-\varepsilon )$$

In which, $\varepsilon$ represents the porosity determined from:

11 $$\varepsilon ={\frac{{\rho }_{i}-{\rho }_{f}}{{\rho }_{i}-{\rho }_{a}}}$$

where, ${\rho }_{i}$ and ${\rho }_{a}$ stand for densities of ice and air, respectively. Nield et al. suggested a geometric average mean shown by k_g_ for the most porous media^22^:

12 $${k}_{g}={k}_{a}^{\varepsilon }{k}_{i}^{1-\varepsilon }$$

Negrelli adapted above equation for frosted media by considering k_f_ = k_g_ as follows:

13 $$log{\frac{{k}_{f}}{{k}_{i}}}={\text{log}}\;a+b \cdot \varepsilon\;log\;{\frac{{k}_{a}}{{k}_{i}}}$$

where, a and b are optimum fitted parameter on experimental data.

## Methodology

### Data collection

Investigation on the available experimental works on frost topic illustrates that while there are some works on frost thermal conductivity in the literature, the studies concentrating on the parallel surface channels are limited. Therefore, two comprehensive works for parallel surfaces are only available. In this study, a total number of 57 actual frost thermal conductivity values in terms of time (t), air velocity (u_a_), relative humidity (Φ_a_), air temperature (T_a_), wall temperature (T_w_), and frost porosity (ε) has been collected^16,21^. The statistical analysis on the collected databank has been reported in Table 1. In addition, dataset can be found in Table S1 of “Supplementary file S1”.

**Table 1:** The details of experimental frost thermal conductivity.

Parameter	Maximum	Minimum	Standard deviation	Mean
t (min)	230	30	64.56	122.61
ε	0.94	0.65	0.07	0.85
T_w_ (°C)	− 7	− 23	5.86	− 15
T_a_ (°C)	15	− 3.5	5.58	6.06
Φ_a_	80	70	3.28	77.19
u_a_ (m/s)	2.2	1.2	0.28	1.31

### Gaussian process regression

This approach which is known as a non-parametric approach can model and simulate different arbitrary complex systems. The advantage of this approach, including flexibility of algorithm in the description of the uncertainty causes attraction of researchers in prediction topics to this algorithm^23^. GPR model has potential to capture the uncertainty. For instance, in the regression process, this method gives a distribution of predicted values instead of only one predicted value. Additionally, employing this method gives users, ability to add specifications and knowledge about the shapes of models by using various forms of kernel function. In this algorithm, time series are modeled by employing a mean function of m(x) and covariance function of (CovF) k(x, x′) as described below:

14 $$ y = f\left( x \right)\sim N\left( {m\left( x \right), k\left( {x,x^{\prime}} \right)} \right) $$

In the above description, input and output of the training set are shown by x and y respectively. The latent variable of the algorithm is described by f(x). In most of the applications, it is common to choose the mean function of discussing equation to zero. The similarity between input variables is described by CovF which is a vital parameter in this algorithm because for the data points which have the similar value of x are more probable to obtain a same value of output. The present work utilizes various types of kernel function, which are described in the following:

- Exponential
  15 $$ k\left( {x,x^{\prime}} \right) = \theta _{1}^{2} {\text{exp}}\left( { - \frac{r}{{\theta _{2} }}} \right) $$
  16 $$r=\sqrt{{\left( {x,x^{\prime}} \right)}^{T} \left(x-{x}^{{{\prime}}} \right)}$$
- Squared exponential:
  17 $$k(x,{x}^{{{\prime}}})={\theta }_{1}^{2}{\text{exp}} \left(-{\frac{{d}^{2}}{2{\theta }_{2}^{2}}} \right)$$
  In the above equation, θ_1_ and θ_2_ represent hyper-parameters which should be optimized. The Euclidean distance of ${x}^{{{\prime}}}$ and x is shown by d.
- Matern
  18 $$k\left(x,{x}^{{{\prime}}}\right)={\frac{1}{\Gamma \left(\nu \right){2}^{\nu -1}}}{\left({\frac{\sqrt{2\nu }r}{l}}\right)}^{\nu }{K}_{\nu }\left({\frac{\sqrt{2\nu }r}{l}}\right)$$
  In this equation, *K*_*v*_ stands for modified Bessel function, and *ν* is a positive parameter.
- Rational quadratic
  19 $$k\left(x,{x}^{{{\prime}}}\right)={\theta }_{1}^{2}\left(1+{\frac{{r}^{2}}{2\propto {\theta }_{2}^{2}}}\right)$$

In which, $\propto$ is a positive parameter of covariance.

To train this algorithm, the hyper-parameters of kernel function can be estimated based on minimization of the negative log marginalized likelihood (NLML):

20 $$NLML=-{\text{log}}\left(p\left(y|x,\theta \right)\right)=-{\frac{1}{2}}{\text{log}}\left|K+{\sigma }_{n}^{2}I\right|-{\frac{1}{2}}{y}^{T}{\left(K+{\sigma }_{n}^{2}I\right)}^{-1}y-{\frac{n}{2}}{\text{log}}(2\pi )$$

This optimization process leads to the calculation of unknown θ. The minimization process of prediction of a parameter can be expressed as below:

21 $$\widehat{\theta }=\underset{\theta }{\text{argmin}}-{\text{log}}(p\left(y|x,\theta \right))$$

To optimize the NLML, the off-the-shelf optimization methods have been employed because of being a convex function. After that, the prediction distribution of the testing phase is shown as follows:

22 $${f}_{*}|x,y,{x}_{*}\sim N(\overline{{f}_{*}},cov\left({f}_{*}\right))$$

23 $$\overline{{f}_{*}}=m\left({x}_{*}\right)+K\left(x,{x}_{*}\right){\left(K\left(x,x\right)+{\sigma }_{n}^{2}I\right)}^{-1}(y-m\left(x\right))$$

24 $$cov\left(\overline{{f}_{*}}\right)=K({x}_{*}{,x}_{*})-K\left({x}_{*},x\right){\left(K\left(x,x\right)+{\sigma }_{n}^{2}I\right)}^{-1}K(x,{x}_{*})$$

where, $cov\left(\overline{{f}_{*}}\right)$ and $\overline{{f}_{*}}$ are the estimation uncertainty and estimation results, respectively. When m(x) = 0, the mean of GPR prediction distribution is a linear function of y for training phase in Eq. (10). Therefore, the mean of GPR prediction distribution is determined as below:

25 $$\overline{{f}_{*}}=K\left(x{,x}_{*}\right){\left(K\left(x,x\right)+{\sigma }_{n}^{2}I\right)}^{-1}y={W}_{GPR}y$$

**Figure 1:**
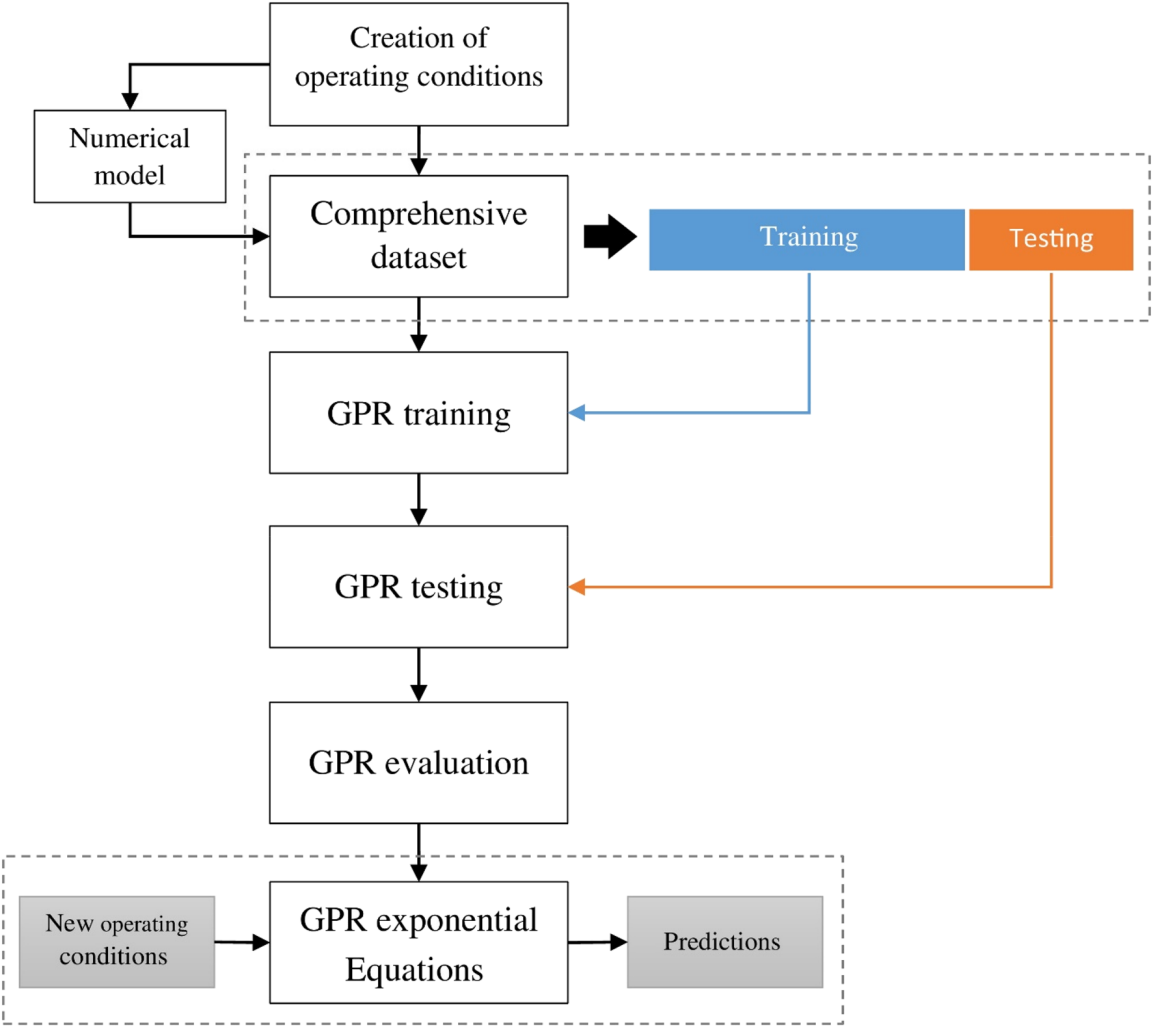
Flowchart for GPR model.

In the above equation, W_GPR_ stands for the weighting matrix^24,25^. A scheme of the GPR model is shown in Fig. 1. This model has been performed in Matlab software.

### Evaluation of the accuracy of the collected databank

A suspected data or outlier is known as a data point that has different behavior in comparison with other data points. The suspected data dominantly refers to the existing errors in the experimental method. The existence of suspected data in databank concludes to incorrect estimations for suggested models. Therefore, the identification of suspected data can improve the integrity and efficiency of suggested models. Leverage method proposes a solution to improve the quality of databank. This algorithm can search for suspected or outlier data point by defining the Hat matrix as follows:

26 $$H=U{({U}^{T}U)}^{-1}{U}^{T}$$

In this equation, U points to a matrix of i*j dimensional. The number of model parameter and training points are shown by i and j. The outlier testing process includes a parameter, namely, critical leverage limit, which is used to identify the suspected data from the remainder of the data. The mentioned limit is determined as follows^26–28^:

27 $${H}^{*}=3(j+1)/i$$

**Figure 2:**
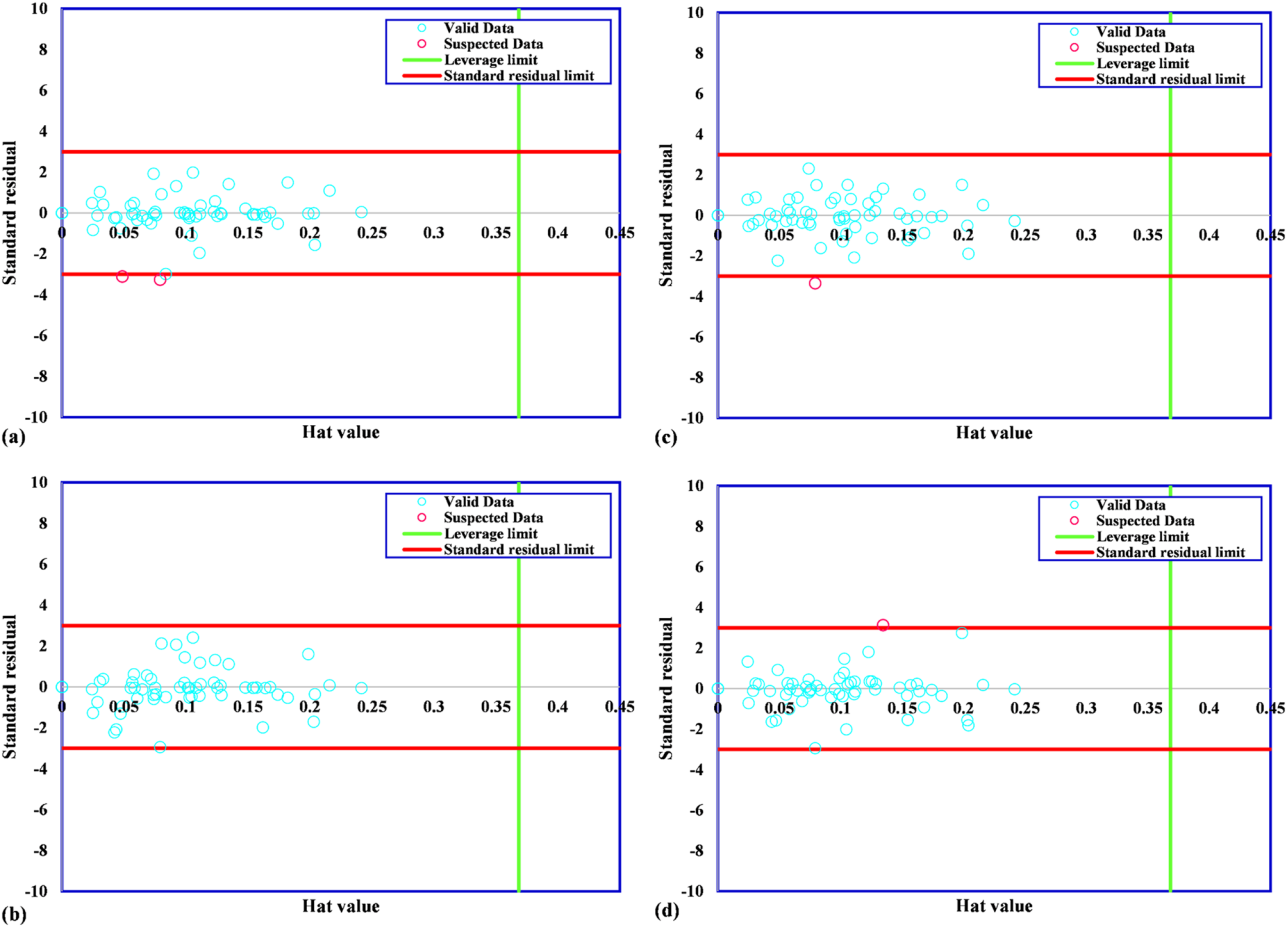
Detection of outliers for GPR models including (**a**) exponential, (**b**) squared exponential, (**c**) rational quadratic, (**d**) matern.

In order to assess the accuracy of frost thermal conductivity databank, the William’s plot concept has been used. As can be seen in Fig. 2, the standardized residuals are depicted versus hat values. In this illustration, the reliable zone for the utilized dataset is defined by the bounded area between standardized residuals of − 3 to 3 and critical leverage limit. The William plot for the thermal conductivity databank shows that the extracted data points are mostly reliable. The number of suspected data are 2, 0, 1, and 1 for Exponential, Squared Exponential, Rational quadratic, and Matern GPR models, respectively. Therefore, the dataset is suitable for training and testing models.

## Results and discussion

### Sensitivity analysis

Identification of the effect of input variables on the thermal conductivity of frost has vital importance for researchers and engineers for proposing a precision model. To achieve this end, a sensitivity analysis for the frost thermal conductivity has been performed. The relevancy factor for each input variable is determined as below^29,30^:

28 $$r={\frac{\sum_{i=1}^{n}({X}_{k,i}-\overline{{X}_{k}})({Y}_{i}-\overline{Y})}{\sqrt{\sum_{i=1}^{n}{{(X}_{k,i}-\overline{{X}_{k}})}^{2}\sum_{i=1}^{n}{({Y}_{i}-\overline{Y)}}^{2}}}}$$

In this equation, ${Y}_{i}$ and ${X}_{k,i}$ are the output and input. $\overline{Y}$ and $\overline{{X}_{k}}$ stand for means of outputs and inputs.

**Figure 3:**
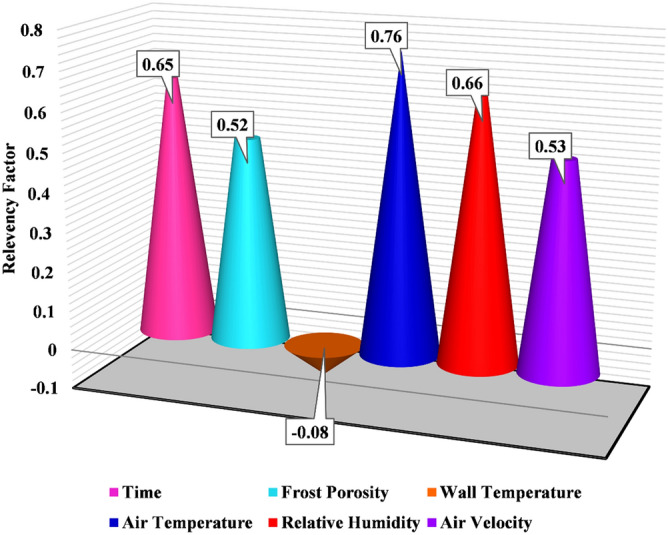
The sensitivity analysis for frost thermal conductivity.

Figure 3 illustrates the impact of each parameter on the frost thermal conductivity. In the discussing method, as the absolute value of r of an input variable is higher, its effectiveness on thermal conductivity is higher and vice versa. In addition, the positive value shows that the discussing input variable has a straight relationship with the frost thermal conductivity. The results of sensitivity analysis express that air temperature with a positive r value of 0.76 is the most effective variable for the frost thermal conductivity determination, which has a straight relationship with the frost thermal conductivity. As can be seen in this figure, wall temperature with an r value of − 0.08 is the least effective parameter in the variation of the thermal conductivity. In addition. The negative sign of relevancy for this variable explains that as the wall temperature increases, the frost thermal conductivity decreases. On the other hand, the other input variables, including frost porosity, time, relative humidity, and air velocity, have increasing relationships with the discussing target value.

### Modeling results

In this section, a numerous number of attempts has been carried out to investigate the performance of proposed models for prediction of the frost thermal conductivity. There are two main ways to assess the performance of the proposed models. These ways are using matching parameters and graphical comparisons. The matching parameters have been used to determine a match between predicted and actual frost thermal conductivity are Mean relative error (MRE), Root mean square error (RMSE), Standard deviations (STD), Mean squared error (MSE), and R-squared (R^2^).

The mathematical parameters have been determined and reported in Table 2 for training, testing and overall dataset. As shown in this table, GPR models with Matern, Exponential, Squared Exponential, and Rational Quadratic kernel functions have R^2^ values of 0997, 0.995, 0.990 and 0.987, respectively. Additionally, the low values of different error parameters such as MRE, RMSE, MSE, and STD in the training phase for the suggested GPR models express that they have been trained in acceptable accuracy. Generally, the accuracy of the training phase of the model has vital importance, but the performance of the model in prediction of unseen frost thermal conductivity point is not ignorable.

**Table 2:** The statistical parameters of developed GPRs.

	R^2^	MRE (%)	MSE	RMSE	STD
**GPR (rational quadratic)**					
Train	0.987	4.08134	0.00005	0.00707	0.00471
Test	0.983	4.67914	0.00004	0.00636	0.00439
Total	0.986	4.22817	0.00005	0.00636	0.00460
**GPR (exponential)**					
Train	0.995	2.82505	0.00002	0.00444	0.00334
Test	0.990	3.82609	0.00003	0.00554	0.00436
Total	0.993	3.07092	0.00002	0.00554	0.00359
**GPR (squared exponential)**					
Train	0.990	3.71123	0.00004	0.00618	0.00475
Test	0.985	3.95804	0.00004	0.00603	0.00419
Total	0.989	3.77185	0.00004	0.00603	0.00458
**GPR (matern)**					
Train	0.997	1.83752	0.00001	0.00335	0.00257
Test	0.994	2.38225	0.00001	0.00370	0.00282
Total	0.996	1.97131	0.00001	0.00370	0.00261

**Figure 4:**
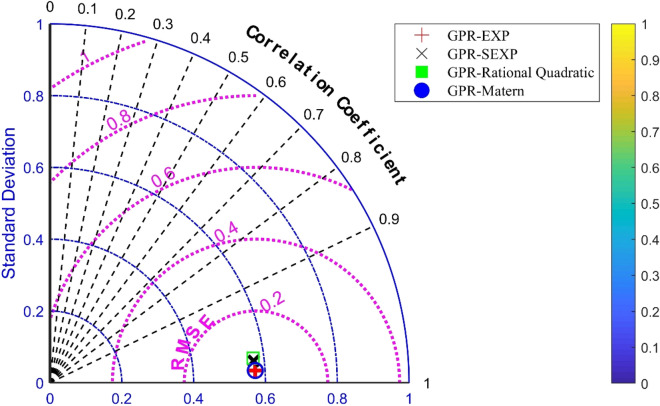
Taylor plot for GPR models.

Therefore, the present models have been evaluated for the testing phase. As can be seen, the most accurate model in prediction of the unseen dataset of frost thermal conductivity is GPR model containing Matern kernel function in which, R^2^, MRE, MSE, RMSE, and STD are 0.994, 2.38225, 0.00001, 0.0037, and 0.00282, respectively. In addition, the Taylor plot has been depicted for discussing models in Fig. 4. In this illustration, the performances of models have been assessed based on R^2^, RMSE, and standard deviation values. According to this depiction, all the models have correlation coefficients of higher than 0.9, RMSE values of lower than 0.2, and standard deviations of lower than 0.6 which express the high quality of suggested models in prediction of the frost thermal conductivity.

**Figure 5:**
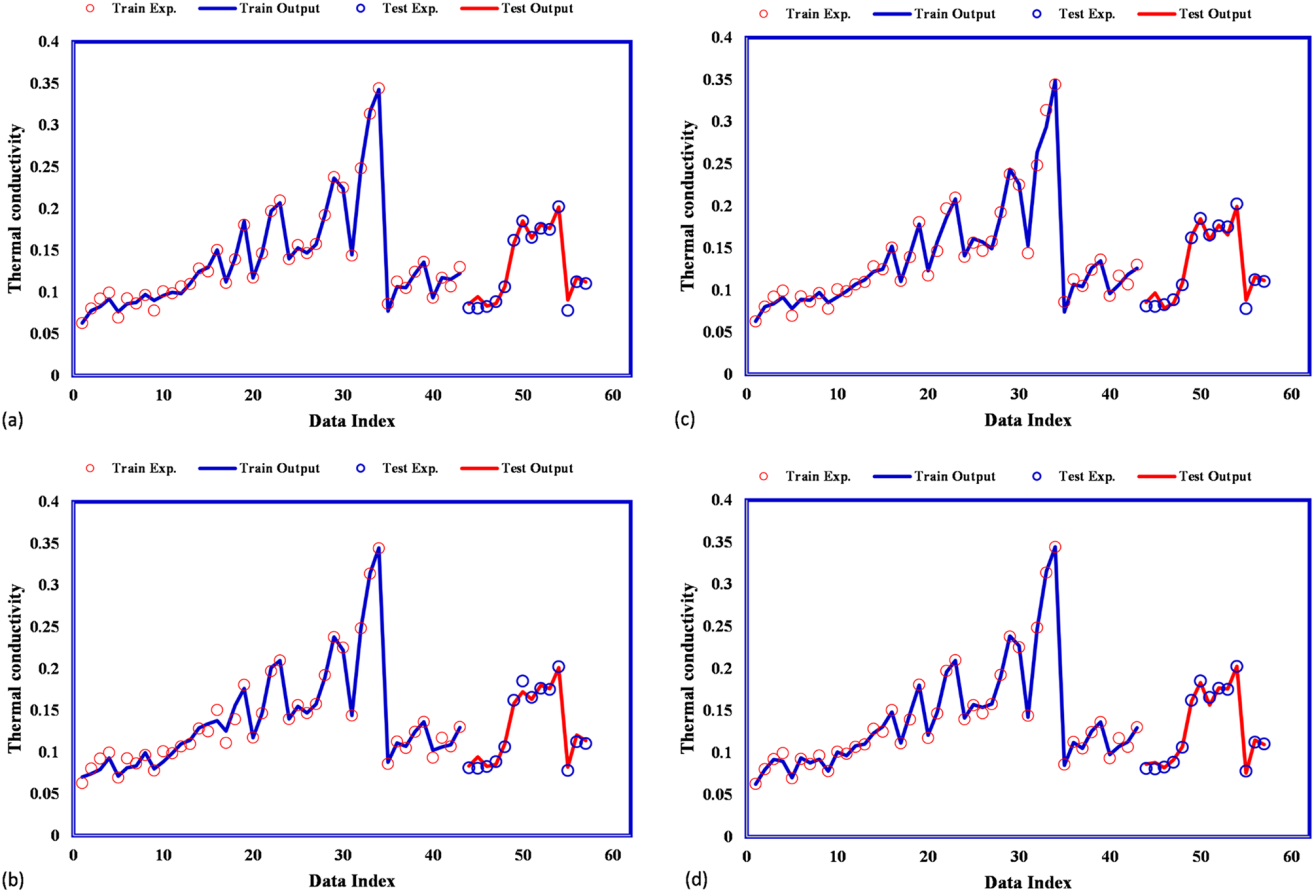
Comparison of model outputs and experimental values for (**a**) exponential, (**b**) squared exponential, (**c**) rational quadratic, (**d**) matern.

On the other hand, the simultaneous comparisons of estimated and experimental frost thermal conductivity values for these models are shown in Fig. 5. Comparing GPR models and experimental thermal conductivity shows the excellent agreement between different GPR models and actual thermal conductivities.

**Figure 6:**
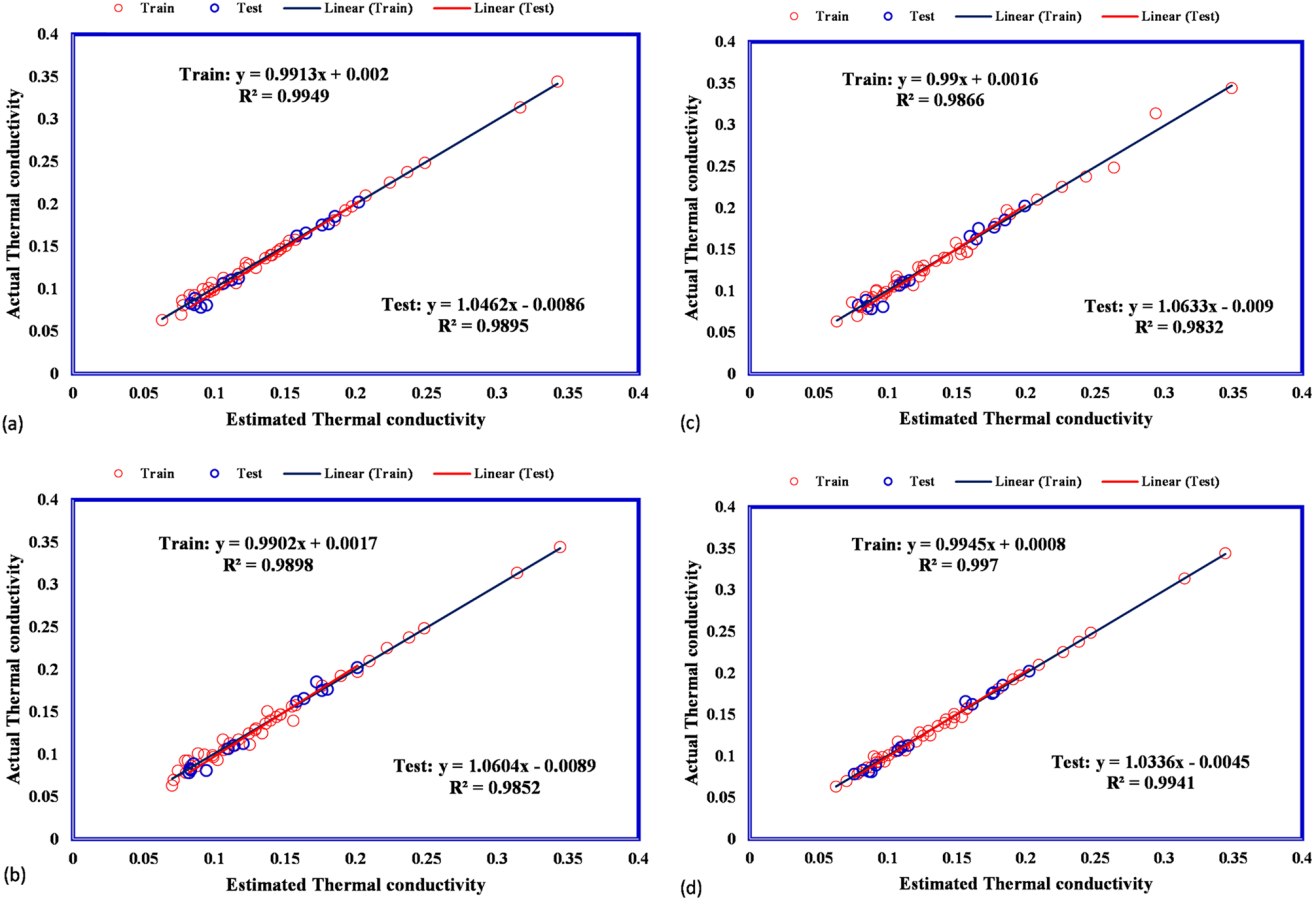
Cross plots for (**a**) exponential, (**b**) squared exponential, (**c**) rational quadratic, (**d**) matern.

**Figure 7:**
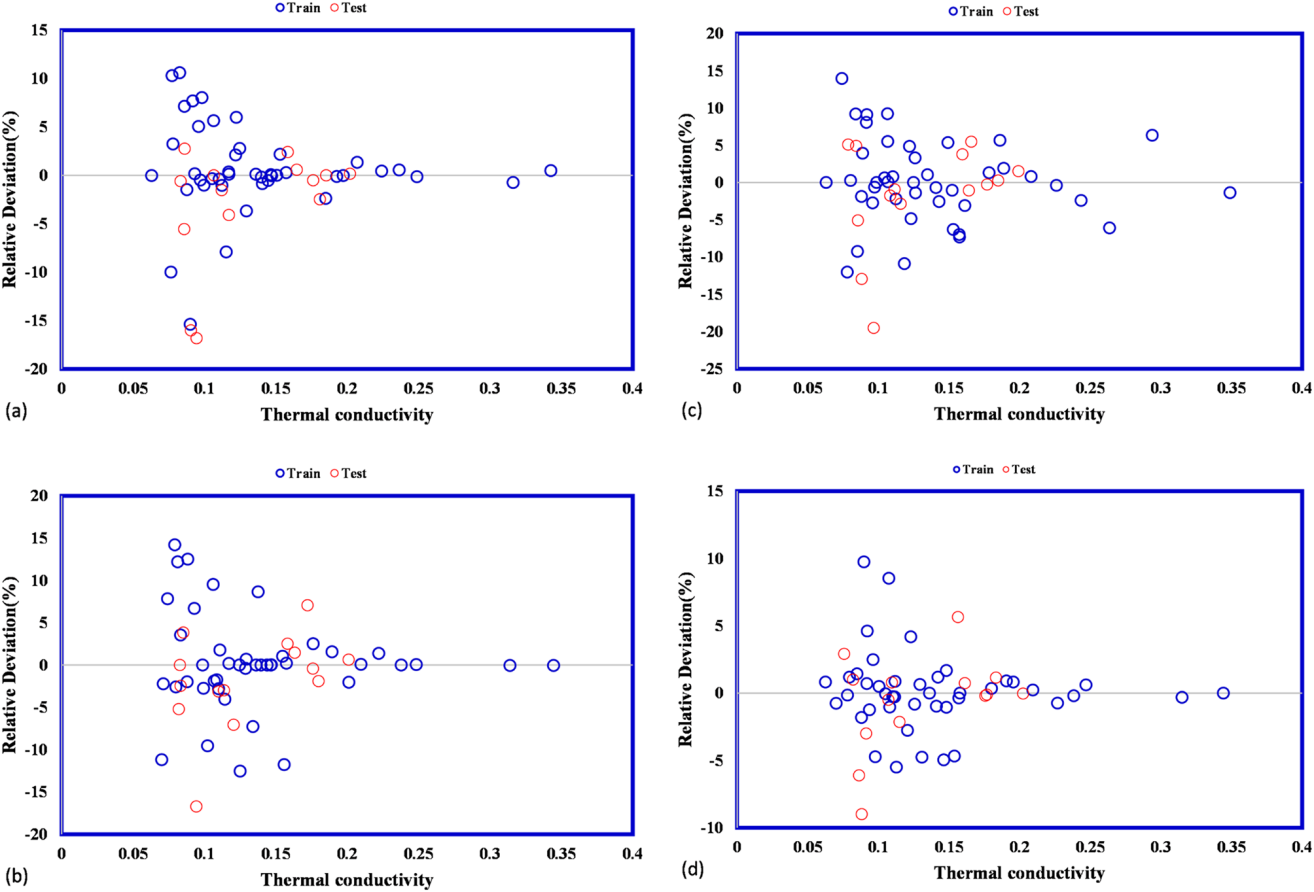
Comparison of model outputs and experimental values for (**a**) exponential, (**b**) squared exponential, (**c**) rational quadratic, (**d**) matern.

The predicted thermal conductivity covers experimental thermal conductivity for all suggested models accurately. Therefore, the GPR models show reliable performance in the prediction of thermal conductivity. Next, the cross plots for different suggested models are illustrated in Fig. 6. These plots for four types of GPR model show that all predicted frost thermal conductivity are located to their actual values so that the fitting lines on them have a similar equation to the bisector line of the first quarter approximately. The bisector line is a measurement for the accuracy of suggested models. As the points locate closer to the bisector line, the accuracy of suggested model is higher. After that, the relative deviations between estimated frost thermal conductivity and actual value are calculated and shown in Fig. 7 for all proposed models. The absolute deviation points for different kernel functions of Exponential, Squared exponential, and Rational quadratic are lower than 20% whereas for Matern kernel function they are lower than 10%.

According to the discussions, suggested GPR models have enough abilities in the prediction of frost thermal conductivity. After that, it is worthy to ensure that the proposed GPRs have enough accuracy in estimation of the frost thermal conductivity. Therefore, the available correlations in the literature have been employed to compare with the suggested models. Table 3 shows the statistical parameters, including MRE, MSE, and RMSE of all available correlations. In this table, among nine studied correlations, N–J–H model has the best prediction with MRE = 11.80, MSE = 5.04e − 04, and RMSE = 0.02 obtained in comparison with actual frost thermal conductivity dataset. These results express that all developed GPR models for prediction of thermal conductivity have better performance the previous published correlations.

**Table 3:** The statistical parameters of previous correlations.

	B–R–S	N–H	N–J–H	O–A	S	Y–S	L–L–K	S–H–K–M	K–J–L
MRE	14.66	20.27	11.8	45.26	15.02	18.27	48.65	55.75	146.09
MSE	9.91E − 04	9.91E − 04	5.04E − 04	3.90E − 03	5.59E − 04	9.70E − 04	3.30E − 03	7.96E − 03	4.35E − 02
RMSE	0.03	0.03	0.02	0.06	0.02	0.03	0.06	0.09	0.21

## Conclusion

In the present work, GPR models including four types of kernel function have been implemented for determination of frost thermal conductivity in terms of time, air velocity, relative humidity, air temperature, wall temperature, and frost porosity. Evaluating the proposed models by the collected thermal conductivity databank have concluded to the high degree of match between forecasted and actual frost thermal conductivity values. According to the mathematical and visual comparisons, the GPR models have excellent ability in the determination of thermal conductivity. On the other hand, the available correlations in the literature have been employed to compare with proposed models. The GPR models have better performance over the utilized databank than employed correlations. It is worthy to mention that a larger experimental databank can improve the accuracy of proposed models during the training phase. Therefore, it is suggested in future works more comprehensive experimental databank should be collected. In addition, the sensitivity analysis on the aforementioned parameters expresses that all input parameters except wall temperature have a straight relationship with the frost thermal conductivity. On the other hand, air temperature is known as the most effective variable. Due to these discussions, the current study can be helpful research for engineers dealing with a refrigeration system and heat exchanger. The present work gives engineers an accurate way to predict the behavior of heat exchanger and refrigeration systems by little knowledge in artificial intelligence methods.

## Supplementary Information

Supplementary Table S1.
