# Supplementary material for: An insight into the estimation of frost thermal conductivity on parallel surface channels using kernel based GPR strategy
Source: Sci Rep. 2021 Mar 30;11:7203. doi: 10.1038/s41598-021-86607-2 (PMC8009876; doi:10.1038/s41598-021-86607-2)
Supplement: Supplementary file 1 — Supplementary Table S1. [file 41598_2021_86607_MOESM1_ESM.pdf]

**An Insight into the Estimation of Frost Thermal Conductivity on Parallel Surface Channels Using Kernel based GPR Strategy**

Xuejun Zhou1, Fangyuan Zhou2,* , Maryam Naseri 3,*
1College of Physics and Electronic Information, Yan'an University, Yan'an Shaanxi 716000, China
2College of Physics and Electronic Information, Yan'an University, Yan'an Shaanxi 716000, China
3Department of Chemical Engineering, Faculty of Engineering, Golestan University, Aliabad Katoul, Iran
*Corresponding authors: zhoufangyuan02@163.com (F. Zhou) and naaseri1375@gmail.com (M. Naseri)

**Table S1:** The dataset used for modeling

| Inputs | | | | | | Output |
|---|---|---|---|---|---|---|
| Time | Frost Porosity | Wall Temperature | Air Temperature | Relative Humidity | Air Velocity | Frsot Thermal Conductivity |
| 45.00 | 0.943 | -23.00 | -3.5 | 80.00 | 1.2 | 0.0631 |
| 90.00 | 0.923 | -23.00 | -3.5 | 80.00 | 1.2 | 0.0806 |
| 135.00 | 0.903 | -23.00 | -3.5 | 80.00 | 1.2 | 0.0925 |
| 180.00 | 0.888 | -23.00 | -3.5 | 80.00 | 1.2 | 0.0814 |
| 230.00 | 0.863 | -23.00 | -3.5 | 80.00 | 1.2 | 0.0996 |
| 45.00 | 0.937 | -23.00 | -1 | 80.00 | 1.2 | 0.0697 |
| 90.00 | 0.901 | -23.00 | -1 | 80.00 | 1.2 | 0.0926 |
| 135.00 | 0.891 | -23.00 | -1 | 80.00 | 1.2 | 0.0865 |
| 180.00 | 0.864 | -23.00 | -1 | 80.00 | 1.2 | 0.0809 |
| 225.00 | 0.851 | -23.00 | -1 | 80.00 | 1.2 | 0.0967 |
| 45.00 | 0.927 | -23.00 | 5 | 80.00 | 1.2 | 0.0829 |
| 90.00 | 0.9 | -23.00 | 5 | 80.00 | 1.2 | 0.0781 |
| 135.00 | 0.876 | -23.00 | 5 | 80.00 | 1.2 | 0.1011 |
| 180.00 | 0.859 | -23.00 | 5 | 80.00 | 1.2 | 0.0988 |
| 230.00 | 0.859 | -23.00 | 5 | 80.00 | 1.2 | 0.107 |
| 45.00 | 0.907 | -15.00 | 4 | 75.00 | 1.2 | 0.0887 |
| 90.00 | 0.866 | -15.00 | 4 | 75.00 | 1.2 | 0.11 |
| 135.00 | 0.84 | -15.00 | 4 | 75.00 | 1.2 | 0.1284 |
| 180.00 | 0.829 | -15.00 | 4 | 75.00 | 1.2 | 0.1249 |
| 225.00 | 0.82 | -15.00 | 4 | 75.00 | 1.2 | 0.1507 |
| 45.00 | 0.9 | -15.00 | 10 | 80.00 | 1.2 | 0.1066 |
| 90.00 | 0.859 | -15.00 | 10 | 80.00 | 1.2 | 0.1112 |
| 135.00 | 0.83 | -15.00 | 10 | 80.00 | 1.2 | 0.1624 |
| 180.00 | 0.791 | -15.00 | 10 | 80.00 | 1.2 | 0.1395 |
| 225.00 | 0.77 | -15.00 | 10 | 80.00 | 1.2 | 0.1808 |
| 45.00 | 0.877 | -15.00 | 15 | 80.00 | 1.2 | 0.1175 |
| 93.00 | 0.814 | -15.00 | 15 | 80.00 | 1.2 | 0.1854 |
| 125.00 | 0.782 | -15.00 | 15 | 80.00 | 1.2 | 0.1467 |
| 180.00 | 0.778 | -15.00 | 15 | 80.00 | 1.2 | 0.1973 |
| 225.00 | 0.696 | -15.00 | 15 | 80.00 | 1.2 | 0.2099 |
| 45.00 | 0.903 | -7.00 | 5 | 70.00 | 1.2 | 0.1398 |
| 92.00 | 0.86 | -7.00 | 5 | 70.00 | 1.2 | 0.1565 |
| 135.00 | 0.843 | -7.00 | 5 | 70.00 | 1.2 | 0.1658 |
| 180.00 | 0.825 | -7.00 | 5 | 70.00 | 1.2 | 0.147 |
| 230.00 | 0.781 | -7.00 | 5 | 70.00 | 1.2 | 0.1766 |
| 45.00 | 0.869 | -7.00 | 10 | 75.00 | 1.2 | 0.1578 |
| 90.00 | 0.818 | -7.00 | 10 | 75.00 | 1.2 | 0.1754 |
| 135.00 | 0.795 | -7.00 | 10 | 75.00 | 1.2 | 0.1925 |
| 180.00 | 0.744 | -7.00 | 10 | 75.00 | 1.2 | 0.2379 |
| 225.00 | 0.741 | -7.00 | 10 | 75.00 | 1.2 | 0.2253 |
| 45.00 | 0.834 | -7.00 | 15 | 80.00 | 1.2 | 0.144 |
| 90.00 | 0.775 | -7.00 | 15 | 80.00 | 1.2 | 0.2024 |
| 135.00 | 0.724 | -7.00 | 15 | 80.00 | 1.2 | 0.2486 |
| 180.00 | 0.696 | -7.00 | 15 | 80.00 | 1.2 | 0.314 |
| 229.00 | 0.645 | -7.00 | 15 | 80.00 | 1.2 | 0.3444 |
| 30.00 | 0.922 | -15.00 | 4 | 75.00 | 1.2 | 0.0862 |
| 60.00 | 0.899 | -15.00 | 4 | 75.00 | 1.2 | 0.0781 |
| 90.00 | 0.872 | -15.00 | 4 | 75.00 | 1.2 | 0.1129 |
| 120.00 | 0.853 | -15.00 | 4 | 75.00 | 1.2 | 0.1126 |
| 30.00 | 0.919 | -15.00 | 4 | 75.00 | 1.7 | 0.1051 |
| 60.00 | 0.891 | -15.00 | 4 | 75.00 | 1.7 | 0.1105 |
| 90.00 | 0.876 | -15.00 | 4 | 75.00 | 1.7 | 0.1246 |
| 120.00 | 0.849 | -15.00 | 4 | 75.00 | 1.7 | 0.1363 |
| 30.00 | 0.919 | -15.00 | 4 | 75.00 | 2.2 | 0.0934 |
| 60.00 | 0.895 | -15.00 | 4 | 75.00 | 2.2 | 0.1174 |
| 90.00 | 0.87 | -15.00 | 4 | 75.00 | 2.2 | 0.1069 |
| 120.00 | 0.85 | -15.00 | 4 | 75.00 | 2.2 | 0.1303 |
